# Design and evaluation of chalconeimine derivatives as α-amylase inhibitors

**DOI:** 10.6026/97320630015523

**Published:** 2019-07-31

**Authors:** Prithivirajan Balu, Jebastin Sonia Jas, Marimuthu Govindaraj

**Affiliations:** 1 Research and Development Centre, Bharathiar University, Coimbatore-641046, India; 2 Department of Chemistry, IFET College of Engineering, Villupuram-605108, India; 3 Department of Chemistry, Swami Dayananda College of Arts and Science, Manjakkudi-612610,Tiruvarur District, India

**Keywords:** Molecular docking, diabetes, alpha-amylase, hydrogen bond, hydrazone, chalcone

## Abstract

Alpha-amylase is a known target for type II diabetes. Therefore, it is of interest to design α-amylase inhibitors based on hydrazone scaffold. The structure of these hybrids was confirmed by spectroscopic analysis (IR, ^1^H-and ^13^C NMR). All the compounds have potential inhibitory properties as shown by in vitro α-amylase inhibition activity. The compound 5-((1Z,3Z)-3-(benzo[d][1,3]dioxol-5-yl)-3-((2-chloropyridin-3- yl)imino)prop-1-en-1-yl)-2-(difluoromethoxy)phenol(4a) in 100 µg/mL concentration showed a high inhibition of 85.23%. In vitro α-amylase inhibition was further supported by docking studies of compound against the active site of pig pancreatic α-amylase (PDB ID: 3L2M). Docking studies revealed that the bonding interactions found between the compound and human pancreatic α-amylase are similar to those responsible for α-amylase inhibition by acarbose.

## Background

Diabetes is a multi-factorial disorder of the pancreas, in which the pancreas fails to perform its function to produce insulin hormone properly in the body. It involves multiple disorders like hyperglycemia, glycosuria and abnormal metabolism of lipids, carbohydrates and proteins [1,2].This affects the human body at physiological, physical and social level. It has been known as the 3rd leading cause of death in humans along with other diseases such as cancer, cerebro-vascular and heart Hypoglycemic medication is helps to lower the blood sugar level in body or treat the other severe symptoms and complications of diabetes mellitus [2]. The side effects of these medications include extreme hypoglycemia, liver cell injury, lactic acidosis, digestive discomfort, permanent neurological deficit, headache, dizziness and even death [3,4]. The basic challenge in curing diabetes is to maintain blood glucose level close to normal levels [5-10]. These therapies are used as mono therapy or in combination for optimal control of glycemia [11-14].As mentioned before that these drugs are normally expensive and come with side effects. These drugs have their limitations due to low pharmacokinetic properties, secondary failure rates and relative bad effects [15-21]. Thus, there is a need for efficient class of compounds to reduce the side effects. Molecular docking is a competent tool for novel micro molecule drugs discovery for targeting protein. This study has been carried out in order to identify effective, selective and efficient antidiabetic Lead compound and its analogues.

Chalcone is a class of open-chain flavonoids that is not only biosynthesized by plants but also can be prepared synthetically. The simplest chalcone can be prepared by an aldol condensation between a benzaldehyde and an acetophenone in the presence of base [22-24]. Hydrazones of chalcones have shown a wide variety of pharmacological effects, including anti-inflammatory and anticancer activities [25-29].Despite the comprehensive biological studies on chalcones, reports on their anti-diabetic activity are limited [30]. Significant advances have been made in the past few years in the isolation and preparation of several hydrazones of chalcones derivatives.

## Methodology

### Chemistry:

Thin layer chromatography (TLC) was used to examine the progress of the reaction. Open glass vessels were used to make a decision for the dissolving on outstanding softening mechanical assembly and were uncorrected. H1 and 13C atomic enticing reverberation (H1 proton magnetic resonance and 13CNMR) spectra were recorded on Bruker Avance II four hundred proton magnetic resonance spectroscope (400 MHz) at 298K, in correct deuterated dissoluble. Concoction move were accounted for as δ (ppm) with relation to tetra methyl silane (TMS) within allowable limit. Infrared spectra (IR) were recorded as KBr pellet on Shimadzu FT-IR spectroscope.

### Preparation of AC-CdO-TiO2 nonocomposite material by precipitation method:

AC-CdO-TiO2 nanocomposite material was synthesized by precipitation method. Initially cadmium acetate dihydrate (0.4 M) were dissolved in anhydrous ethanol solution beaker A. 0.4m citric acid and tetra isopropyl orthotitanate were in ethanol is taken as another solution beaker B and Activated carbon (AC) were dissolved in anhydrous ethanol solution beaker C, the solution A and solution C is added to Solution beaker B and stirred well. Then to thise 2 drops NH4OH is added at room temperature under vigorous stirring until the precipitate was formed. The obtained precipitate was washed with water and ethanol. Then the precipitate was collected and dried in oven at 100oC for 12 hrs. The resulting powder was finally calcinated at 5000 C at 4 hrs.

### General procedure for the Synthesis of (E)-1-(4-(difluoromethoxy)-3-hydroxyphenyl)-3-phenylprop-2-en-1-one(3):

4-(difluoromethoxy)-3-hydroxybenzaldehyde 1 (0.02 mol) and 1- (benzo[d][1,3]dioxol-5-yl)ethanone 2 (0.02 mol) were dissolved in 30 ml of alcohol. To this reaction mixture 40% NaOH (10 ml) and AC-CdO-TiO2 nanoparticles catalyst (0.003 g), in ethanol (5 mL) were added. TLC followed the progress of the reaction. After completion of the reaction, the mixture was filtered to remove the catalyst and the filtrate was taken in ether, washed with water and dried over anhydrous sodium sulfate. Removal of solvent gave the crude product which was recrystallized from methanol to obtain the pure compounds.m.p:960C; M.F: C17H12F2O5;M.W:334.

### General procedure for the Synthesis of 5-((1Z, 3Z)-3- (benzo[d][1,3]dioxol-5-yl)-4-(substituted pyridin-2-yl)buta-1,3- dien-1-yl)-2-(difluoromethoxy)phenol (4a-e):

(E)-1-(4-(difluoromethoxy)-3-hydroxyphenyl)-3-phenylprop-2-en-1- one 3 (0.01mol) and substituted aniline (0.01 mol) was dissolved in ethanol (20 ml). To this mixture AC-CdO-TiO2 nano particles was added and it was refluxed for 3 hrs. On cooling and dilution with ice-cold water, a solid mass separated out. It was re-crystallized from ethanol.

### Docking studies:

X-ray crystal structures of pig pancreatic alpha-amylase (PDB Id: 3L2M) were retrieved from the Protein Data Bank [31]. To put together the receptor for docking studies, co-crystallized ligand and water molecules were eliminated. At the same time polar hydrogen atoms and Kollman-united costs have been protected by the DNA Gyrase receptor. The essential pdb and pdbqt documents of ligands and Pig pancreatic alpha-amylase receptor were prepared for the AutoDock 4.2 software [32]. The usual docking protocol was applied in the AutoDock Vina in PyRx 0.8 software [33]. The docking results were analyzed using Discovery Studio 4.0 (Accelrys, Inc. San Diego, CA 92121, U.S.).

### Inhibition assay for α-Amylase activit:

A stock solution of 10 mg/10 mL concentration was prepared using DMSO solvent. Activity of amylase was assayed with different concentrations (50, 100, 200 µg/mL) of sample with control and reagent solution without test sample was used as control. Starch solution (1% w/v) or (0.5% w/v) was prepared by stirring and boiling 0.5 g of soluble potato starch in 50 mL of deionized water for 15 minutes. The enzyme solution (1 unit/mL) was prepared by mixing 100 mg in 100 mL of 20 mM sodium phosphate buffer (pH 6.9). The color reagent was a solution containing 96 mM 3,5- dinitrosalicylic acid (DNSA) (20 mL), 5.31 M sodium potassium tartrate in 2 M NaOH (8 mL) and deionized water (12 mL). Acarbose was used as a standard at the concentration of 1mg/mL. 100 µl of test solution and 100 µL of enzyme solution were mixed in viols and incubated at 25°C for 30 min. To this mixture 100 µL of color reagent was added and the mixture was heated on water bath at 85°C for 15 min. Further, the reaction mixture was removed from water bath, cooled and absorbance value determined at 595 nm. Individual blanks were prepared for correcting the background absorbance. Control experiment was conducted in the same manner by replacing the drug sample with 1 mL DMSO. Inhibition percentage of α- amylase was calculated by the formula [34]. Enzyme activity was calculated and percentage of inhibition is ((Control-Test)/100) x 100.

## Results and Discussion:

**Figure 1:**
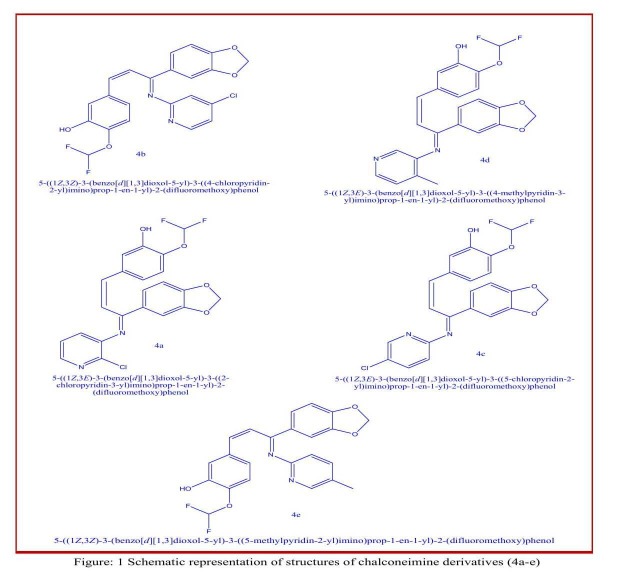
Schematic representation of structures of chalconeimine derivatives (4a-e)

Table 1 show all the physical data like color, molecular formula, molecular weight, solubility, melting point, of synthesized compounds. The IR frequencies of compounds 4a-e is shown in Table 2 in which the C=N stretching frequency appear at 1586-1667 cm-1. Aromatic (CH) stretching frequencies appear at 3084-3093cm-^1^ and stretching frequency observed at 1625-1666cm^-1^ C=O group present in the derivatives. The 1H NMR chemical shift values of compound (4a-e) given in Table 3. The singlet observed in the range 6.30-6.60ppm is due –CH_2_ methylene proton of 3',4'- methylenedioxy acetophenone moiety proton. The singlet observed at 7.41-7.49ppm is due –CH proton of –CHF_2_ moeity. The signals appearing 7.14-8.38ppm are obviously due to aromatic protons. The five chalconeimine derivatives (4a-e) shown in Figure 1a were taken for docking studies. These compounds are synthesized and their structures have been determined by IR,^1^H and ^13^CNMR spectroscopy.

## In vitro α-amylase inhibition:

All the synthesized compounds (4a-e) and standard drug were explored for their in vitro α- amylase inhibition studies at different concentrations (50, 100, 200µg/mL) as shown in the Table 4. All the compounds showed good % inhibition of α-amylase when compared with standard drug acarbose. Compound 4b and 4d were found to be more potent among all the synthesized compounds when explored at the concentration of 50µg/mL. Compound 4d shows 76.58% inhibition followed by 4b with 77.18% inhibition. There was a significant rise in % inhibition when concentration has been changed to 100µg/ml from 50µg/mL. Among all, 4b shows 81.35% inhibition followed by 4a which showed 85.23% inhibition at 100µg/ml. Inspired by the results obtained at 100µg/mL concentration, all the synthesized compounds were further screened for there in vitro α-amylase inhibition at 200µg/mL. All compounds exhibited a linear rise in % inhibition.

## Docking studies:

**Figure 2:**
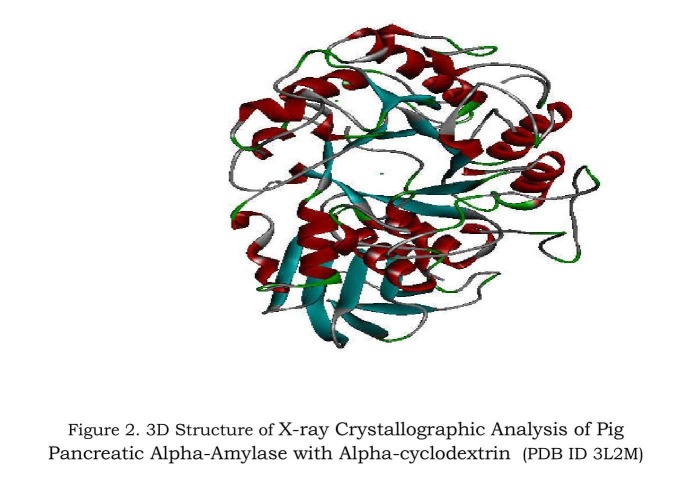
3D Structure of X-ray crystallographic analysis of pig pancreatic alpha-amylase with alpha-cyclodextrin (PDB ID: 3L2M)

Interactions between inhibitors and active site of the target protein can be explored using molecular docking studies. The above results showed that all the synthesized molecules were stronger inhibitors of alpha-amylase as compared to acarbose. Therefore, for ascertaining the binding conformation and interactions responsible for the activity, docking simulation of compound 4a and 4d was performed against active site of pig pancreatic alpha-amylase (PDB ID: 3L2M). Ligands taken for the docking studies are shown in Figure 1a. Pig pancreatic alpha-amylase protein is considered as target protein for this study. Its structure was taken from RCSB Protein Data Bank (PDB) with PDB ID: 3L2M as shown in Figure 2. Target protein has their active sites where the compound shows maximum number of interaction with protein. The complete dataset was docked and found to bind at the same active site position. Amino acids are intimately involved in the binding ligand to protein and form a complex. The residue that is significant for binding interaction and thus comprising the binding pocket of target protein are shown in Table 4. Docking studies reveled that these amino acids present in the target proteins pocket involves in the binding interaction with the selected compounds.

**Figure 3:**
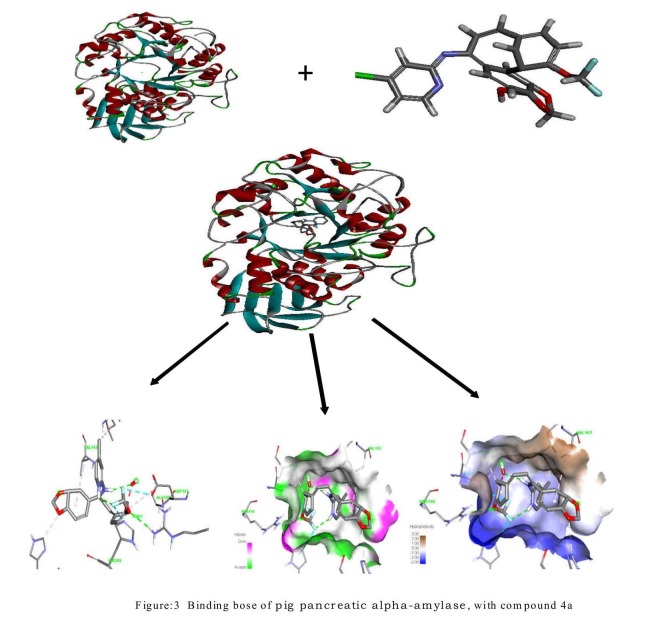
of pig pancreatic alpha-amylase, with compound 4a

These complex structures reveal essential interactions between the inhibitor and the protein and these interactions are taken as the reference for the hydrazone derivative (4a-e). The co-crystallized ligand are forms hydrogen bond interaction with the residues GLY 309, GLN 302, ARG 346, ASP316, ARG 267) (Figure 5) which are present within the ATP binding pocket. The ligand is also further stabilized by a number of hydrophobic contacts with the residues. The five hydrazone derivatives (4a-e) shown in Figure 1a were taken for docking studies. These compounds are synthesized and their structures have been determined by IR, 1H and13CNMR spectroscopy. The docking studies clearly reveal that some of these compounds bind efficiently to the enzymes of pig pancreatic alphaamylase. Binding score of autodock 4.2 varies between -7.8 to -8.9 for compounds 3a-g tested for pig pancreatic alpha-amylase (Table 5) Out of the five hydrazone derivatives analyzed, compound 4b and 4d forms the best interaction with pig pancreatic alphaamylase. The compound 4a and 4d has the highest binding score of -8.9 and - 8.7. The fluorine, oxgen atom on hydrazone compound forms hydrogen bond with the hydrogen atom of ALA 198, ARG 195, and HIS 299 of pig pancreatic alpha-amylase (Figure 3 and Table 6).

**Figure 4:**
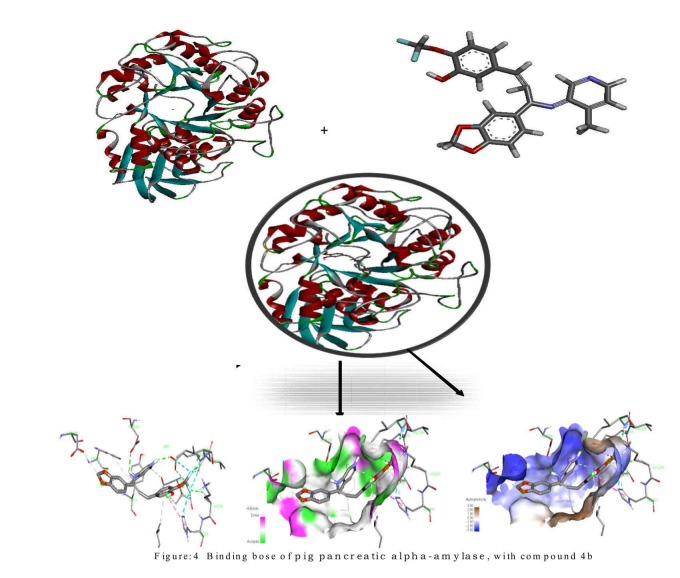
Binding of pig pancreatic alpha-amylase, with compound 4b

**Figure 5:**
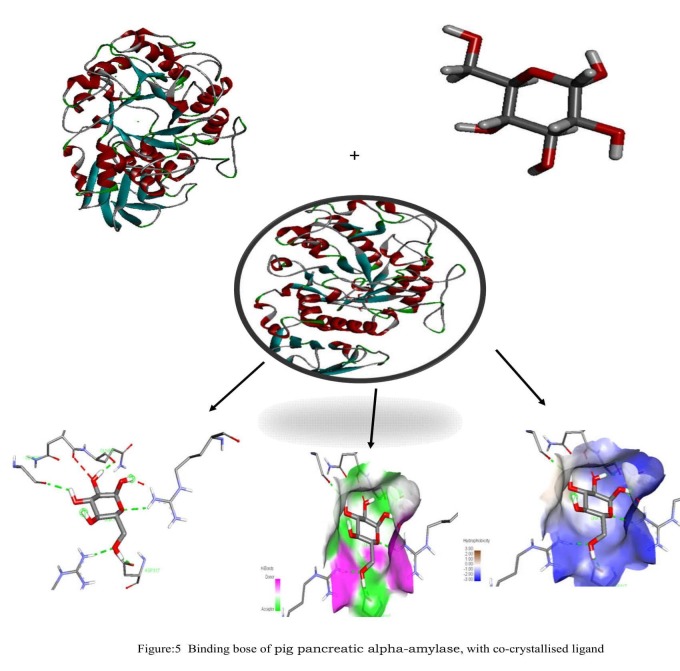
Binding of pig pancreatic alpha-amylase, with cocrystallized ligand

Compound 4d having a binding score of -8.9 makes hydrogen bonds with the active site residue ASP 300, GLU 233, LYS200 and ILE 235 of enzyme (Figure 4).Re-docking of the inhibitor from the co-crystallized complex structure (Figure 5) of pig pancreatic alphaamylase resulted in a binding score of -7.8, which is comparable to the scores found for compound 4b and 4d (Table 5). The re-docked conformation of co-crystallized ligand (Figure 2) resembles the conformation of the hydrazone derivative (compound 4b and 4d respectively).

## Conclusion

We describe the synthesis and evaluation of five hydrazone derivatives as α-amylase inhibitors. The structures of all synthesized compounds were confirmed by elemental and spectroscopic analysis (IR, 1H and 13C-NMR). The biological potential of synthesized compounds was investigated through in vitro α-amylase inhibition activity. The results showed that some of the synthesized compounds exhibited significant inhibitory activities. The compound 5-((1Z,3Z)-3-(benzo[d][1,3]dioxol-5-yl)-3- ((4-chloropyridin-2-yl)imino)prop-1-en-1-yl)-2-(difluoromethoxy) phenol (4b) in 100 µg/mL concentration showed remarkable inhibition of 81.35%. Docking studies of compound 4a-e were performed against active site of pig pancreatic alpha amylase (PDB ID: 3L2M). It has been revealed from docking studies that the bonding interactions found between 4b and 4d Rwith pig pancreatic α-amylase are similar to those responsible for α-amylase inhibition by acarbose.

## Figures and Tables

**Table 1:** Physical data of various synthesized compounds

Compound	Color	Mol. Formula	Mol. weight	Solubility	Melting point (°C)
4a	Yellow	C_22_H_15_C_l_F_2_N_2_O_4_	444	Ethanol	157
4b	Yellow	C_22_H_15_C_l_F_2_N_2_O_4_	444	Ethanol	133
4c	Yellow	C_22_H_15_C_l_F_2_N_2_O_4_	444	Ethanol	148
4d	Pale Yellow	C_23_H_18_F_2_N_2_O_4_	424	Ethanol	128
4e	Pale Yellow	C_23_H_18_F_2_N_2_O_4_	424	Ethanol	118

**Table 2:** Data from IR spectra of chalconeimine derivatives (4a-e)

Compounds	FREQUENCY cm-1				
	C=O	C=N	Ali C-H	CH=CH	ARO C-H
4a	1666	1597	2966	1452	3089
4b	1645	1586	2924	1425	3084
4c	1645	1589	2924	1448	3084
4d	1625	1586	2926	1452	3093
4e	-	1667	2924	1450	3088

**Table 3:** Data from 1H NMR spectra of hyrazone derivatives (4a-e)

Compounds	0	CHF_2_	Aromatic protons
4a	6.30 (2H,singlet)	7.48 (1H,singlet)	7.43-8.36 (11H, multiplet)
4b	6.60 (2H,singlet)	7.47(1H,singlet)	7.47-7.95 (11H, multiplet)
4c	6.55 (2H,singlet)	7.49 (1H,singlet)	7.43-8.38 (11H, multiplet)
4d	6.58 (2H,singlet)	7.46 (1H,singlet)	7.28-7.96 (11H, multiplet)
4e	6.55 (2H,singlet)	7.41 (1H,singlet)	7.14-8.38 (11H, multiplet)

**Table 4:** alpha-amylase inhibition activity of compounds 4a-e

Compound	Concentration (µg/mL)	% Inhibition
4a	50	70.84
	100	85.23
	200	86.84
4b	50	77.18
	100	81.35
	200	83.64
4c	50	54.82
	100	68.58
	200	73.34
4d	50	76.58
	100	75.03
	200	77.84
4e	50	50.12
	100	69.87
	200	71.93
Acarbose	50	56.69
	100	63.85
	200	69.78

**Table 5:** Binding energy of docked compounds (4a-e)

Compound	4a	4b	4c	4d	4e	Co-ligand
Binding energy	-8.9	-8.9	-8.3	-8.7	-8.5	-7.8

**Table 6:** Binding interactions of docked compounds

Compound	Type of interaction	Between	Distance	Type of interaction	Between	Distance	Type of interaction	Between	Distance
4a	Hydrogen bon	NH-0 (GLC 701)	3.04	Halogen	F-0 (GLU 233)	2.99	pi-pi interaction	TYR 62	4.57
		H-0 (GLU 233)	2.53						
4b	Hydrogen bon	NH-O (ALA 198)	2.72	Halogen	F-OD1(ASP 197)	3.65	Alkyl Interaction	VAL 163	5.23
		o-NH (ARG 195)	2.86				Alkyl Interaction	LEU 165	4.09
		NH-F (HIS 299)	2.55		F-NE2(HIS299)	3.31	Alkyl Interaction	ALA 198	5.35
4c	Accptor -aceptor	o-o2 (GLC 701)	2.83	Halogen	F-OD2 (ASP 356)	3.15	Alkyl Interaction	ILE 235	3.99
	Charge -change	N-OD2 (ASP 300)	5.53				Alkyl Interaction	LYS 200	3.82
		N-OD1 (ASP 191)	5.41				Pi-Alkyl	HIS 201	4.82
		N-OE1 (GLU233)	5.06						
4d	Hydrogen bon	NH-oD2 (ASP 300)	2.44	Halogen	F-CD(GLU 233)	3.62	Pi-Alkyl	TYR 62	4.29
		NH-OE1 (GLU 233)	2.28		F-O(GLU 233)	3.44	Pi-Alkyl	TRP 58	4.57
		NH-F (LYS200)	2.54		F-C (ILE 235)	3.52	Pi-Alkyl	ALA 198	5.2
		NH-F (ILE 235)	2.22		F-NE2((HIS 201)	3.69	Pi-Alkyl	LEU 162	4.82
							pi-pi interaction	HIS 201	4.81
4e	Hydrogen bon	-----		Halogen	F-NE2 (HIS 299)	3.2	Pi-Alkyl	VAL 163	5.36
					F-OD1(ASP 197)	3.45	Pi-Alkyl	TRY 62	4.48
					F-OE2(GLU 233)	3.14			
					F-OE1((GLU 233)	2.93			
Co ligand	Hydrogen bon	H-0 (GLY 309)	2.28	Accptor -aceptor	0-0 (GLN 302)	2.98	-----		
		H-0 (GLN 302)	2.01	Donar -Donal	H-H (ARG 346)	2.35			
		O-H (ARG 346)	2.35						
		H-OD1 (ASP316)	2.16						
		O-H (ARG 267)	2.33
